# Interactions of Neutrophils with the Polymeric Molecular Components of the Biofilm Matrix in the Context of Implant-Associated Bone and Joint Infections

**DOI:** 10.3390/ijms242317042

**Published:** 2023-12-01

**Authors:** Davide Campoccia, Stefano Ravaioli, Rasoul Mirzaei, Gloria Bua, Maria Daglia, Carla Renata Arciola

**Affiliations:** 1 Laboratorio di Patologia Delle Infezioni Associate all’Impianto, IRCCS Istituto Ortopedico Rizzoli, Via di Barbiano 1/10, 40136 Bologna, Italy; davide.campoccia@ior.it (D.C.); stefano.ravaioli@ior.it (S.R.); gloria.bua@ior.it (G.B.); 2 Venom and Biotherapeutics Molecules Laboratory, Medical Biotechnology Department, Biotechnology Research Center, Pasteur Institute of Iran, Tehran 1316943551, Iran; rasul.micro92@gmail.com; 3 Department of Pharmacy, University of Napoli Federico II, Via D. Montesano 49, 80131 Naples, Italy; maria.daglia@unina.it; 4 International Research Center for Food Nutrition and Safety, Jiangsu University, Zhenjiang 212013, China; 5 Laboratory of Immunorheumatology and Tissue Regeneration, Laboratory of Pathology of Implant Infections, IRCCS Istituto Ortopedico Rizzoli, Via di Barbiano 1/10, 40136 Bologna, Italy; 6 Department of Medical and Surgical Sciences (DIMEC), University of Bologna, Via San Giacomo 14, 40126 Bologna, Italy

**Keywords:** neutrophils, immune response, periprosthetic infections, orthopedic implants, implant failure, bacterial biofilms, extracellular polymeric substances

## Abstract

In the presence of orthopedic implants, opportunistic pathogens can easily colonize the biomaterial surfaces, forming protective biofilms. Life in biofilm is a central pathogenetic mechanism enabling bacteria to elude the host immune response and survive conventional medical treatments. The formation of mature biofilms is universally recognized as the main cause of septic prosthetic failures. Neutrophils are the first leukocytes to be recruited at the site of infection. They are highly efficient in detecting and killing planktonic bacteria. However, the interactions of these fundamental effector cells of the immune system with the biofilm matrix, which is the true interface of a biofilm with the host cells, have only recently started to be unveiled and are still to be fully understood. Biofilm matrix macromolecules consist of exopolysaccharides, proteins, lipids, teichoic acids, and the most recently described extracellular DNA. The latter can also be stolen from neutrophil extracellular traps (NETs) by bacteria, who use it to strengthen their biofilms. This paper aims to review the specific interactions that neutrophils develop when they physically encounter the matrix of a biofilm and come to interact with its polymeric molecular components.

## 1. Introduction

Bacterial infections are largely recognized as one of the most serious complications that can affect implant materials and eventually determine prosthetic failure [1,2]. Key elements for the success of the invading microorganisms, usually opportunistic pathogens living as saprophytes on human epithelia and mucosal membranes, are the presence of a foreign body (the biomaterial implant) and the ability of bacteria to adhere and colonize its surface, producing protective biofilms.

Once established on the biomaterial surface, bacteria in biofilms are tolerant to medical treatments and elude the host immune response [3]. Thus, life in a biofilm emerges as a fundamental factor, which makes biomaterial-associated infections a very critical clinical problem that can often be solved uniquely by the removal of the infected implant and its replacement.

Immune elusion relies on a variety of mechanisms not exclusively in connection with the biofilm phase. For instance, pathogens such as *Staphylococcus aureus* have been found to be capable of eluding host defenses simply by invading host tissue cells and surviving intracellularly [4]. Even more, under in vivo clinical conditions, *S. aureus* was found to be capable of infiltrating the very thin canaliculi (thinner than the diameter of the bacterium itself) of live cortical bone [5] and so reaches niches that are hidden and not accessible to the activity of immune scavenger phagocytic cells. Apart from these elusion strategies based on the physically hiding of bacteria, pathogens such as staphylococci can skew opsonization and consequent leukocytes phagocytosis by expressing specific virulence factors. For instance, *S. aureus* can prevent complement activation through a series of different mechanisms, including by binding IgG immunoglobulins through *S. aureus* protein A (SpA) and staphylococcal binder of immunoglobulin (Sbi); by inhibiting the classical pathway (C1q complement component binding by the collagen adhesin, CNA); by cleaving the C3 complement component (C3 cleavage by aureolysin); by inhibiting C3 cleavage into C3b (Sbi); by blocking C3 (extracellular adherence protein, Eap, and staphylococcal complement inhibitor, SCIN) as well as C5 convertases (extracellular fibrinogen-binding protein, Efb); by cross-linking C3b to fibrinogen (Efb); and by activating plasminogen on the bacterial surface (staphylokinase, SAK) [6]. Cell wall proteins have been shown to provide crucial opportunities for bacteria to interact with the host and are essential for the colonization of host tissue and even for survival in host cells. Noteworthy is the contribution of *S. aureus* protein A (SpA) to immune evasion by binding the Fc region of IgG molecules and impairing their opsonizing activity. The interaction between SpA and the Fc region of IgG results in the blocking of IgG hexamerization through a competitive binding to the Fc–Fc interaction interface on IgG monomers [7]. The block of IgG hexamerization would in turn prevent the formation of the (IgG)_6_:C1q complexes, thus hindering downstream complement activation on the surface of *S. aureus*. However, SpA was known to block IgG-mediated phagocytosis even in the absence of complement. Only recently, the mechanism implicated has been unveiled and SpA was found to block IgG-mediated phagocytosis and killing of *S. aureus* by inhibiting the interaction of IgGs with FcγRs (FcγRIIa and FcγRIIIb, but not FcγRI) and FcRn [8]. Protein A-deficient bacteria are thus more susceptible to neutrophil-mediated uptake and killing in the presence of serum and have reduced virulence.

Many other evasion strategies have been shown in multiple studies, although these are often referring to planktonic bacteria. Nevertheless, understanding them can also help to enlighten neutrophil–biofilm interactions, as it is likely that some evasion mechanisms occur in biofilms as well or could concur to biofilm formation by hiding from the sight of neutrophils; those planktonic bacteria will subsequently organize themselves in a biofilm community. In 2019, Masters et al. [9] described three pathogenically distinct reservoirs of biofilm bacteria in osteomyelitis: glycocalyx on the implant, colonization of osteocyte lacuno-canalicular network, and staphylococcal abscess communities (SACs).

Much remains to be unveiled on the protection from the host immune response gained by bacteria living in a biofilm. In an earlier review, we reported on the ability of pathogens causative of implant infections and their biofilms to hijack immune defenses, thus impairing an efficient eradication of bacteria and inducing the establishment of a chronic osteomyelitis [10]. Recent studies have highlighted the existence of a crosstalk between bacteria and the effector cells of the immune system, neutrophils among them, describing the mechanisms and roles of what is referred to as immunometabolism [11]. For instance, recent studies demonstrated that phagocytosed bacteria stimulate neutrophil itaconate production that suppresses the oxidative burst [12], while biofilm-derived lactate stimulates the production of the anti-inflammatory cytokine IL-10 by neutrophil precursor cells [13].

**Figure 1:**
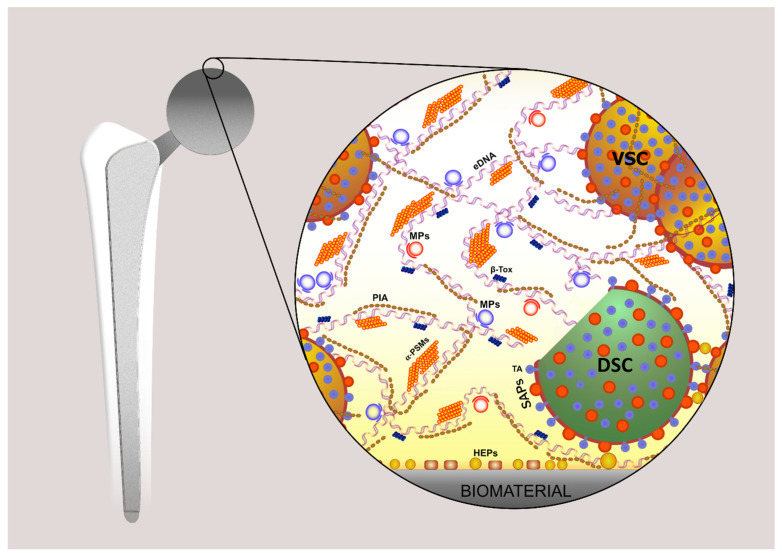
This figure schematically illustrates the complex architecture of a biofilm of *S. aureus* established on the surface of and around a hip prosthesis. It shows, on the left, an infected hip prosthesis and, on the right, a magnified schematic view of the biofilm, revealing its main polymeric components: PIA, an exopolysaccharide, namely the polysaccharide intercellular adhesin; eDNA, extracellular DNA; SAPs, surface-associated proteins (e.g., SasG, clumping factor B, SdrC, Bap, FnBPA, and FnBPB); amyloidogenic proteins (e.g., β-Tox, β-Toxin; PSMs, phenol-soluble modulins such as α-PSM1); MPs, moonlighting proteins (cytoplasmic proteins with a moonlighting role in the biofilm matrix); HEPs, host extracellular matrix proteins; TA, teichoic acids; VSC, viable staphylococcal cell; DSC, dead staphylococcal cell. It must be taken into consideration that, under real in vivo conditions, a further level of complexity is associated with the existence of polymeric substances contributed by the host tissues themselves.

Apart from all the above mechanisms of interaction, neutrophils, once recruited at the site of infection by chemotactic and haptotactic signals, physically meet bacterial biofilms at the interface of orthopedic implants with periprosthetic tissues. Initially conceived as a mere accumulation of exopolysaccharides, the concept of the biofilm extracellular matrix has consistently evolved and emerged as a rather complex molecular architecture (Figure 1), where different categories of extracellular polymeric substances (EPS) mutually interact and assemble [14,15]. EPS currently recognized to take part in the biofilm extracellular matrix architecture include the following molecules: extracellular DNA (eDNA), exopolysaccharides, proteins, teichoic acids, and lipids. eDNA has been found to be a nearly universal component of microbial biofilms of bacterial and even fungal species. Conversely, EPS such as the exopolysaccharides vary for typology and expression across different genera, species, and, occasionally, even bacterial strain types.

The present review precisely explores, brings together, and discusses the currently available information on the specific interactions developed when neutrophils encounter biofilms and sense the EPS molecules of the biofilm matrix. Particular attention is paid to opportunistic bacterial pathogens that most frequently cause biofilm-based orthopedic implant infections, namely staphylococci.

## 2. Influences on Biofilm–Neutrophils Interplay

Implant infections can develop in the early hours after surgery by contaminant bacteria as well as, at a later stage, following hematogenous seeding in the presence of a bacteriemia. Under both circumstances, if planktonic bacteria are not immediately cleared by pre- and intraoperatively administered antibiotics and the host immune defenses, there is a real chance for bacteria to adhere to and colonize the prosthetic surfaces. In this latter circumstance, neutrophils meet a much harder enemy and have lower chances of success. The biofilm matrix is actively produced, assumes the role of a protective shield, and becomes the real physical interface between bacteria and neutrophils. A pronounced diversity in biofilm EPS composition has been observed in different bacterial species but even strain types of the same species [16]. The same bacterial strains themselves have been found to be capable of forming different biofilms under varying environmental conditions [17]. In this connection, it may be important to understand if, to what extent, and how the interactions with each single EPS component can influence neutrophil behavior. There are three possible routes of interaction between neutrophils and biofilm components: (1) interactions with leaching polymeric molecules that can be released from the biofilm and act as chemoattractants or trigger neutrophil polarization/activation; (2) direct interactions with the polymeric components that take part in the biofilm architecture; and (3) interactions mediated by active complement components generated following opsonization of the biofilm surface. Even though over the years many efforts have been made to clarify the mechanisms and the effects of these interactions, much still remains unveiled. In some cases, the results appear contradictory, and doubts remain on the correct interpretation due to the difficulty in perfectly purifying single components [18]. Many investigations have been conducted in vitro and the results might not reflect the more complex in vivo circumstances. Moreover, while in many studies the interactions have been investigated with single purified components, the different structural elements of the biofilm matrix are known to interact with each other forming acid–base, polycationic–polyanionic complexes [14]. The significant interactions that, for instance, could take place by eDNA-exopolysaccharides or eDNA-proteins are largely unknown and span from complement activation to recognition by neutrophils receptors.

In addition to the receptors for components of the complement cascade (C3a receptors, C3aR, and C5a receptors, C5aR), neutrophils express a series of other receptors useful to recognize the presence of invading microbes. Host defenses against infections rely on the effective immune recognition of microbial components with specific motifs (the so-called pathogen-associated molecular patterns, PAMPs), which enable the host to distinguish self- from non-self-molecules. Neutrophils express pattern recognition receptors (PRRs) that are devoted to the recognition of PAMPs such as formylated peptides, lipoteichoic acid, peptidoglycan, and lipoproteins [19]. They include, among others, the receptors for the formylated peptide FPR1 and FPR2, Toll-like receptors (TLRs), nucleotide-binding oligomerization domain-like receptors, and C-type lectin receptors [6]. The paragraphs that follow will review the interactions of neutrophils with each different EPS component.

**Figure 2:**
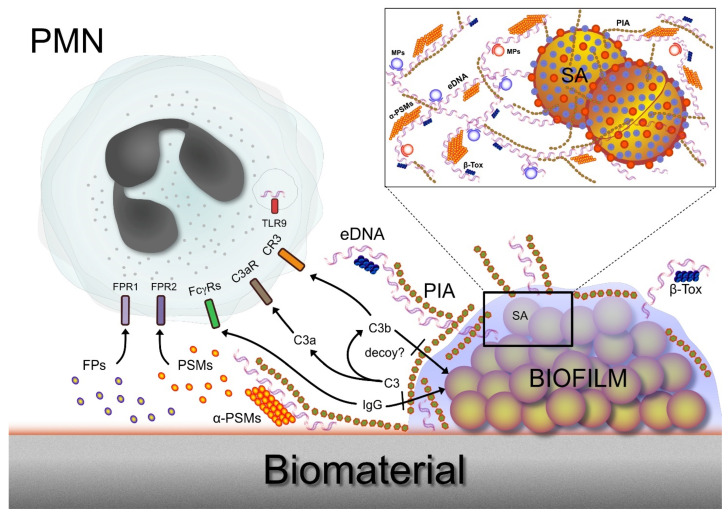
This figure illustrates a polymorphonuclear neutrophil (PMN) that encounters an *S. aureus* biofilm on a biomaterial surface. A series of receptors that are expressed either on the surface or on the membranes of intracellular vesicles enable the PMN to directly recognize specific polymers taking part in the biofilm architecture or indirectly sense complement components generated following the triggering of the complement cascade by biofilm EPS. At the same time, EPS components such as PIA appear to protect bacteria from opsonization and their consequent targeting and phagocytosis by PMNs, as discussed in the next sections. The inset box on the upper right corner shows a magnified schematic view of the biofilm, revealing the complex weaving of extracellular polymeric components of matrix. Legend: amyloidogenic proteins (e.g., β-Tox, β-Toxin; α-PSMs, phenol-soluble modulins such as α-PSM1); C3, complement component 3; C3a, C3a protein formed by the cleavage of complement component 3; C3b, C3b protein formed by the cleavage of complement component 3; C3aR, complement component 3a receptor; C3R, complement receptor 3 (alternatively termed CD11b/CD18); eDNA, extracellular DNA; FPs, formyl peptides; FPR1 and FPR2, respectively, formyl peptides receptor 1 and 2 [20]; FcγRs, Fc receptors for IgG; MPs, moonlighting proteins (cytoplasmic proteins with a moonlighting role in the biofilm matrix); PIA, polysaccharide intercellular adhesin; PSMs, phenol-soluble modulins; SA, *S. aureus* cell; TLR9, Toll-like receptor 9 (expressed in intracellular vesicles).

Figure 2 reports some of the relevant interactions taking place when neutrophils physically encounter *S. aureus* biofilms on the biomaterial surface.

## 3. Extracellular DNA

The present review specifically focuses on the specific interactions taking place when neutrophils encounter biofilms and sense the extracellular polymeric substances that constitute the biofilm matrix. Until a few years ago, it would have been somewhat surprising to see a review on the interactions of the EPS with neutrophils firstly treating eDNA rather than any other biofilm polymeric components. In fact, over the years, eDNA has emerged as a fundamental biofilm matrix component, nearly universally observed across biofilms of different bacterial species [21]. In view of its ubiquity, the study of eDNA has therefore attracted increasing interest not only for the study of clinically relevant biofilms but also, more generally, for assessing the ecological status of different environmental niches. The highly conserved use of eDNA for bacterial adhesion and biofilm formation across species points out to a very early adaptation and repurposing of this molecule during the phylogenesis of prokaryotic evolution.

Initially observed in biofilms of *Pseudomonas aeruginosa*, *Streptococcus intermedius*, and *Streptococcus mutans*, the presence of eDNA has since been confirmed in biofilms of other pathogens such as *Enterococcus faecalis* and staphylococci [14,22]. Bacterial autolysis is the common mechanism by which eDNA is released [23]; nonetheless, even lysis-independent mechanisms have been discovered. *S. aureus* autolysis is mediated by murein hydrolase, while in *Staphylococcus epidermidis*, it is mediated by the autolysin protein AtlE. In *S. aureus*, the crucial role of eDNA in stabilizing biofilm is highlighted by the disaggregating effect of DNase I. Rajendran et al. reported on the contribution of the locus *comEB* to eDNA-dependent biofilm formation in *Staphylococcus lugdunensis* and indicated that *comEB* stimulates biofilm formation via a lysis-independent mechanism of DNA release [24]. Does eDNA only come from bacteria? Alhede et al. used transmission electron and confocal scanning laser microscopy to examine the interaction between biofilms of *P. aeruginosa* and polymorphonuclear neutrophils (PMNs) in a murine implant model. In in vivo biofilm infections by *P. aeruginosa*, the majority of eDNA at the infected site was from the host. Noticeably, they observed a PMN-derived external layer. This PMN-derived eDNA-surrounded biofilm was not observed within biofilm [25]. Interestingly, eDNA can be enzymatically modulated and tailored by bacteria to diverse functions, e.g., biofilm strengthening, nutrient supply, horizontal gene transfer, and interaction with immune defenses [14]. Thus, it turns out to be a very versatile molecule that can be easily repurposed for many tasks.

Bacterial eDNA and other microbial structural motifs are recognized by the innate immune system via the TLRs family of PRRs [26]. Nucleic acids such as DNA are common to eukaryotic and prokaryotic cells and, thus, microbial DNA is a difficult target to recognize from self-DNA [27]. Neutrophils, as other effectors cells of the immune system, recognize bacterial molecules including bacterial eDNA through TLRs. Bacterial DNA differs from eukaryotic DNA due to its higher frequency of unmethylated CpG dinucleotides, and this feature is sensed by a specific Toll-like receptor, TLR9 [28,29].

There is consistent evidence that DNA either released by lysed bacteria within leukocytic phagosomes or in the form of eDNA within the biofilm matrix acts as a proinflammatory molecule. Under both circumstances, the involvement of TLR9 has been reported. This receptor is expressed in intracellular vesicles (e.g., endoplasmic reticulum, endosomes, lysosomes, and endolysosomes), where the recognition of microbial nucleic acids takes place [30]. TLR9, following the recognition of eDNA within the intracellular vesicles, generates the type 1 interferon response to biofilms. In *P. aeruginosa* biofilms, an amyloidogenic protein, namely amyloid curli, has been suggested to form molecular complexes with eDNA and act as a carrier of eDNA to the neutrophils’ intracellular vesicles compartments. TLR2, another member of the Toll-like receptors expressed on the outer surface of neutrophils, has been suggested to recognize the amyloid curli–eDNA complexes and mediate the transfer to the intracellular vesicles [31,32].

Fuxman Bass et al. found that the degradation of eDNA with DNase I reduced both the ability of *P. aeruginosa* biofilm to evoke the release of proinflammatory cytokines by neutrophils and the upregulation of neutrophil activation markers [33]. In the same experimental model, enzymatic degradation of eDNA also reduced the number of bacteria phagocytosed per neutrophil and, noticeably, the production of neutrophil extracellular traps. Together, these experimental findings support the idea that eDNA favors neutrophil activation and enhances the innate immune response towards biofilms. Nonetheless, apart from the broadly recognized interaction with TLR9, other experimental work conducted by the same research group suggest the possibility that immobilized bacterial DNA could activate neutrophils through CpG- and TLR9-independent mechanisms. This alternative mechanism of recognition would not require eDNA internalization and would be triggered by DNA molecules consisting of more than ≈170 nucleotides [34]. Interestingly, it has been further hypothesized that long human DNA molecules (for instance, the DNA released from neutrophils following NETosis) may exert a steric hindrance in bacterial DNA binding or bind to TLR9 without causing activation of the transductional pathways [35]. Under these circumstances, host DNA would inhibit the activation induced by bacterial eDNA.

Thus, bacterial eDNA appears as a well-established PAMP, broadly expressed across bacterial species, and for which the signaling pathway has been elucidated. It represents an important proinflammatory component that is capable of eliciting the type 1 interferon response to bacterial biofilms. Conversely, eucaryotic eDNA derived from NETosis or tissue cell death, although binding to TLR9, does not trigger the leukocyte response and is hypothesized to prevent the binding of bacterial eDNA in biofilms.

## 4. Biofilm Exopolysaccharides

Staphylococcal species such as *Staphylococcus epidermidis* and *S. aureus* represent the most prevalent etiological agents causing orthopedic implant-related infections. The polysaccharide intercellular adhesin (PIA), alternatively known as poly-β-(1→6)-*N*-acetylglucosamine (PNAG), is the main exopolysaccharide expressed by both these staphylococcal species. The *ica*ADBC locus, encoding PIA, is always present in *S. aureus* and in the majority of *S. epidermidis* strains, although its expression depends on environmental conditions [36]. Encoded by the *icaB* gene, the surface-attached protein IcaB is responsible for the deacetylation of the poly-*N*-acetylglucosamine molecule, conferring a polycationic charge to the partly deacetylated polymer [37]. In *S. epidermidis*, it has been estimated that deacetylation of the polymer accounts for approximately 15–20% of *N*-acetylglucosamine residues [38].

The mechanisms of modulation of biofilm production as well as the influence of environmental factors are important subjects of research since they deal with the interactions of biofilms with the immune cells. An early debated question was the phase variation of *S. epidermidis*. Ziebuhr et al. ascribed the inhibition of PIA synthesis to the introduction of the insertion sequence element IS*256* in the *ica* locus and suggested that the naturally occurring insertion/excision of IS*256* modulates the expression of the *ica* locus [39]. A subsequent investigation aimed at detecting all genes of the *ica* operon by multiple PCR was carried out on a collection of *S. epidermidis* clinical isolates from periprosthetic infections [40]. The genes of the *ica* locus were found strictly linked to each other, appearing either all simultaneously present or all absent in the genome of a single isolate. In that collection of clinical *S. epidermidis* isolates, the insertion element IS256 was either in phase variants obtained by repeated cultures [39] or in strains subjected to chemical mutagenesis [41], but never in clinical isolates. Therefore, it was not proved to be a naturally occurring mechanism of on/off switch of biofilm production. Kozitskaya et al. observed that bacterial insertion sequence element IS*256* occurs preferentially in nosocomial *S. epidermidis* isolates, in association with biofilm formation and resistance to aminoglycosides [42]. More recently, IS*256* insertion sequence has been shown to prevent biofilm formation also in *Enterococcus faecalis* [43]. A further mechanism of phase variation of poly-*N*-acetylglucosamine in *S. aureus* has been described, involving the expansion and contraction of a tetranucleotide tandem repeat within *icaC* [44]. In recent years, researchers have mainly focused their studies on the influence of environmental conditions (for example, aeration versus oxygen deprivation, availability of free iron, oxidative stress, sequestration of essential nutrients) on staphylococcal growth, transcription of the *ica* gene, and the expression of the intercellular polysaccharide adhesin [45,46,47].

It has been observed for a long time that PIA represents the first factor of the staphylococcal biofilm matrix recognized as protecting bacteria from neutrophils. Vuong et al. demonstrated that phagocytosis and killing by neutrophils was significantly increased in a mutant *S. epidermidis* strain unable to produce PIA in comparison with the PIA-producing wild-type strain [48]. Recently, another study reported that PIA added to planktonic bacteria was capable of significantly reducing opsonic killing. Moreover, the same work demonstrated that the quantity of PIA in the biofilm matrix is sufficient to bind to the opsonic antibodies and subsequently inhibit killing of otherwise susceptible bacteria [49]. The contribution to virulence of PIA in biofilm has also been demonstrated by Kropec et al. in a murine model of systemic infection by *S. aureus*. Bacterial strains defective for the *ica* genes were found to be much more susceptible to antibody-independent opsonic killing involving neutrophils and complement, demonstrating that PIA confers to *S. aureus* resistance to killing mediated by the innate host immune cells [50]. Therefore, on the contrary of eDNA, PIA emerges as an inert biofilm component that appears to play a role in bacterial shielding and, likely, in masking other bacterial and biofilm components that constitute PAMPs recognized by neutrophil receptors such as TLRs. Interestingly, in an isogenic *icaB* mutant strain of *S. epidermidis*, neutrally charged non-deacetylated poly-acetylglucosamine was reported to lose PIA’s typical properties that are functional to bacterial virulence. This strongly supports the importance of the cationic character of PIA [32]. As also discussed by Le et al. (2018) [51], in front of many studies supporting a sheltering activity of PIA from the host innate immune response, some work indicates a potent proinflammatory activity of PIA, mediated by a strong activation of the complement system [52]. The same authors also reported that PIA-based biofilms prevented IgG and C3b deposition as well as neutrophil phagocytosis and bactericidal activity. While these findings warrant further investigations, some possible explanations have been hypothesized [6]. For instance, the highly activated complement on PIA is not deposited in the proximity of the bacterial surface and acts as a decoy for the target [49]. Alternatively, although inducing C3a release, PIA could prevent the C3b deposition on the bacterial surface [53].

*P. aeruginosa* is another pathogen intensively investigated for its ability to produce different morphotypes of polysaccharide-based biofilms. Malhotra et al. (2019) [54] recently reported on the host–microbe interface in cystic fibrosis, reviewing the ability of this pathogen to adapt the biofilm composition during the evolution of the disease. Three main exopolysaccharides have been identified in *P. aeruginosa* biofilms: alginate, Pel, and Psl. They are differentially expressed in adaptation to the changing environmental conditions and/or the stage of the disease. For instance, in late cystic fibrosis, clinical isolates exhibit a rugose small-colony variants [RSCVs] colony morphotype of small, wrinkled appearance. This morphotype is associated with variants that overproduce Psl/Pel with respect to wild-type *P. aeruginosa*. Apart from exhibiting increased resistance to antibiotics, RSCVs show greater protection from neutrophils, ROS, and antimicrobial peptides. The late emergence of mucoid *P. aeruginosa* variants profoundly impacts the clinical status of the CF patient and is characterized by variants with an overproduction of alginate [54].

Rybtke et al. found that extracellular matrix polysaccharides in *P. aeruginosa* biofilms play a role in the response of PMNs toward biofilms. Using different *P. aeruginosa* mutants producing different matrix exopolysaccharides, they found that *P. aeruginosa* biofilms with distinct exopolysaccharide matrix components elicit distinct PMN responses [55]. The authors observed the increased activation of neutrophils (in terms of both the oxidative burst and degranulation) when they encountered an alginate- and Psl-rich biofilm. They speculated that the structure of the alginate- and Psl-rich biofilm matrix might provide the possibility of the PMNs coming into close contact with the bacteria, and so be triggered to respond. The same vigorous response was not observed for alginate- and Pel-rich biofilm. The authors identified in Psl, rather than in alginate, the cause of the vigorous response of neutrophils to alginate- and Psl-rich biofilm. These findings were, however, achieved by using genetically modified strains and not real clinical isolates. There is evidence that, in CF, Psl exerts a protective action on *P. aeruginosa* from host defenses in the early phase that precedes the switch to the alginate-overproducing mucoid phenotype [56].

*Kingella kingae* is a leading cause of joint and bone infections in young children, which has very recently been reported as a potential pathogen for prosthetic joint infection [57], particularly in patients who are immunosuppressed [58]. The pathogenetic mechanisms that enable *K. kingae* to elude host defenses are still largely unknown. Nonetheless, some evidence has been produced on the possible roles of polysaccharides expressed by this pathogen. Muñoz et al. (2018) had earlier reported that two polysaccharidic components, respectively, the *K. kingae* polysaccharide capsule and the exopolysaccharide, have important redundant roles in promoting pathogen survival in human serum by preventing opsonization/complement activation in an in vivo murine model [59]. More recently, it was observed that the polysaccharide capsule and the exopolysaccharide would also act independently and confer protection from ROS production and neutrophil phagocytosis. More in detail, the polysaccharide capsule was found to interfere with the neutrophil oxidative burst response and prevent neutrophil binding of *K. kingae*, although it had no effect on bacterial internalization by neutrophils. Conversely, the *K. kingae* exopolysaccharide involved in biofilm production would inhibit or delay neutrophil phagocytosis and reduce the sensitivity to antimicrobial peptides [60].

Overall, while bacterial eDNA is universally confirmed as a proinflammatory biofilm component determining neutrophils activation, different exopolysaccharides of main pathogenic species emerge as sheltering molecules that not only do not stimulate neutrophil activation, per se, but often protect bacteria from ROS and AMPs, reduce neutrophils chemotaxis, and prevent phagocytosis and bacterial killing. The ability to activate the complement cascade by exopolysaccharides such as PIA and the implications for bacteria survival in biofilms warrant further investigations. Certainly, complement depletion on biofilm surfaces and decoy could provide some interesting interpretations.

Table 1 summarizes the different activities on the immune response that have been documented for biofilm exopolysaccharides expressed by different bacterial species.

## 5. Biofilm Proteins

An extract containing biofilm matrix exoproteins has been shown to induce a protective immune response against *S. aureus* biofilm by activating a humoral immune response and eliciting the production of interleukin 10 (IL-10) and IL-17 in mice [69]. Understanding the contribution of each antigen present in the biofilm matrix to immune response is difficult.

The fact that biofilm EPS isolated from *S. epidermidis* activates neutrophils leading to the release of cytotoxic and bactericidal components, such as lactoferrin, was confirmed by Meyle et al. in 2012. Specifically, the authors suggested that a defined protein fraction was able to activate neutrophils in vitro [70]. A few years later they identified the bacterial heat shock protein GroEL as a possible candidate since depletion of GroEL by immunoadsorption reduced the capacity of EPS to activate neutrophils. Moreover, recombinant GroEL activated oxygen radical production and upregulation of the adhesion protein CD11b and induced the release of DNA from neutrophils [71,72].

The extracellular adherence protein (Eap) is a secreted protein non-covalently attached to the *S. aureus* cell surface that has been implicated in several aspects of *S. aureus* pathogenesis. Stapels et al., in 2014, found that *S. aureus* secretes a family of Eap proteins that specifically block the activity of neutrophil serine proteases (NSP), which are of major importance in the regulation of NET formation, with inhibitory-constant values in the low-nanomolar range. Crystallography analysis revealed that that Eap molecules can occlude the catalytic cleft of NSPs and in vivo studies suggest that Eap proteins promote S. aureus infection [73]. Since Eap is also involved in *S. aureus* biofilm formation [74], it was hypothesized that it can contribute to binding of eDNA. Eisenbeis et al. indeed demonstrated, by using atomic force microscopy, that Eap specifically binds to linearized DNA, mediating its aggregation to prevent extracellular trapping. When neutrophils were incubated with Eap in the presence of PMA to trigger the production of NET, a reduction in the characteristic staining pattern of NETs was observed [75].

A long-debated question was whether the *ica* locus is always needed for biofilm production. Really, in some *S. aureus* strains, the *ica* operon can be deleted without impairing biofilm production, which therefore depend on an *ica*-independent pathway. This alternative mechanism of biofilm synthesis relies on the ability of *S. aureus* to express a variety of adhesion proteins that promote bacteria attachment to and colonization of many different substrates [76]. Among these proteins, the first recognized was the biofilm-associated protein Bap [77]. Numerous other proteins participate in the biofilm matrix (reviewed in [78]). They intervene at different stages of biofilm formation, some of them mediating the first attachment to the biomaterial surfaces filmed by host matrix proteins and others contributing to biofilm accumulation. In *S. aureus*, in addition to Bap, also SasC and SasG, the clumping factor B (ClfB), the serine aspartate, repeat protein, SdrC, the protein A, and the fibronectin/fibrinogen-binding proteins FnBPA and FnBPB have been identified and studied [79]. *S. aureus* can express up to 24 different cell wall proteins, whereas coagulase-negative staphylococci, such as *S. epidermidis* and *S. lugdunensis*, express a smaller number [79]. Foster et al., in their review, underline that, among the biofilm-associated proteins, FnBPs, protein A, and ClfB are widely distributed, while Bap, SasG (which is a homologue of the *S. epidermidis* Aap), and SasC are present only in subsets of isolates. Notably, cell wall proteins are differentially expressed in biofilms compared to their planktonic counterpart [80]. In addition, accumulating biofilm in an *ica*-independent manner is particularly relevant to MRSA, in both hospital-associated and community-associated strains [81].

The *icaADBC* operon is frequently found in biofilm-forming isolates of *S. epidermidis*. Nonetheless, a further mechanism of induction of biofilm production has been highlighted that does not involve *ica* genes, but the genes that control the synthesis of the accumulation-associated protein (Aap), as below, and of the metalloprotease SepA [82]. Both genes are required for Aap-dependent *S. epidermidis* biofilm formation. Indeed, in a recent paper [83], Gomez-Alonso et al. demonstrated the induction of biofilm production by neutrophil proteases in non-biofilm-forming commensal isolates of *S. epidermidis*. More precisely, commensal isolates with icaA−, icaD− and aap+, sepA+ genotypes, which had previously been recognized as non-biofilm-forming, produced biofilms in the presence of neutrophilic proteases such as cathepsin G, cathepsin B, proteinase-3 and MM-9 in neutrophils. Neutrophils do not generate NETs against commensal bacteria but produce proteases that help commensal bacteria in survival [83]. Aap contributes both to the initial attachment phase and to the intercellular connections, with each cell stretching twisted fibrils of truncated Aap to interact with the neighboring cells. The interesting aspect of this mechanism for biofilm formation via Aap (also SasG) cleavage and self-assembly is that Zn^2+^ ions and proteases are released by immune cells at sites of inflammation [84]. Thus, *S. epidermidis*, with few weapons, can produce its biofilm thanks to neutrophils.

FnBPs and other surface proteins promote biofilm accumulation either by homophilic protein–protein interactions or by binding to other ligands on neighboring cells [85]. Moreover, by interacting with integrins of the eukaryotic cell membrane, such as the abundantly expressed α5β1 integrin, FnBPs promote the invasion into non-phagocytic host cells [86]. This mechanism is based on the internalization of *S. aureus* into osteoblasts [87]. This interaction promotes the expression of “TNF-related apoptosis-inducing ligand” (TRAIL). TRAIL induces caspase-8 activation with the consequent apoptosis of osteoblasts. A further mechanism of staphylococcal invasion of osteoblast is mediated by the interaction of staphylococcal protein A (SpA) and tumor necrosis factor receptor-1A (TNFR-1), which is present on the osteoblast surface. This interaction promotes the expression of the “receptor activator of NF kappa B ligand” (RANKL), which in turn promotes osteoclastogenesis [88,89]. Thus, by the apoptotic death of osteoblasts and the recruitment and activation of osteoclasts, bone destruction ensues.

The correlation between phenol-soluble modulins (PSMs) and the formation of amyloid aggregates is a controversial topic. While in *S. aureus* PSMs have a strong tendency to aggregate and to produce amyloids, which contribute to biofilm stability [90], in *S. epidermidis*, all PSMs were reported to have a role in biofilm structuring without, however, forming amyloids. The PSM-dependent biofilm phenotypes displayed in vitro and in vivo by *S. epidermidis* do not match with the PSM amyloid model proposed for *S. aureus* [91]. In fact, the research group of Otto has ruled out that (i) PSMs form amyloids and (ii) amyloid formation plays a role in the major biological functions attributed to PSMs. According to Otto’s experimental results, PSMs can aggregate to form amyloid-like structures, but amyloid formation does not appear a prerequisite for cytolysis. Also, the attachment of PSMs to eDNA and the resulting resistance of eDNA to degradation by DNase may explain the observed biofilm stability-mediating properties of PSMs without the necessity to refer to an amyloid formation. The biofilm stability reported by Schwartz et al. may simply be due to the observed variations in eDNA presence, with eDNA having the well-established role of an in vitro biofilm matrix component rather than that of a “seed” for PSM amyloid formation [92].

In recent years, microbial amyloids were shown to play major roles in microbial physiology and virulence. For example, amyloid fibers assemble on the bacterial cell surface as a part of the extracellular matrix and are considered important to the scaffolding and structural integrity of biofilms [14], contributing to microbial resilience and resistance. Several non-scaffold roles of bacterial amyloid proteins have been recently reviewed, including the following: bridging cells during collective migration, acting as regulators of cell fate, as toxins against other bacteria or against host immune cells, and as modulators of the hosts’ immune system [93].

The production of pore-forming leukocidal toxins (e.g., alpha-hemolysin, Hla; gamma-hemolysins HlgAB and HlgCB; leukocidin ED, LukED; leukocidin AB/GH, LukAB/GH; and Panton–Valentine leukocidin, PVL) [94] and of phenol-soluble modulins directly targets the viability of human leukocytes. *S. aureus* biofilms have been reported by Bhattacharya et al. to rapidly skew neutrophils toward neutrophil extracellular trap (NET) formation through the combined activity of leukocidins PVL and HlgAB [95]. The authors reported that, by eliciting this response, *S. aureus* can persist, as the antimicrobial activity of released NETs is ineffective at clearing biofilm bacteria. Two different winning strategies were claimed to facilitate *S. aureus* biofilm survival: (1) LukAB would enhance survival and, thus, persistence of biofilm-grown *S. aureus* in the presence of neutrophils; and (2) the expression of the nuclease Nuc would facilitate the degradation of NET DNA and the survival of trapped *S. aureus*, while enabling dissemination of biofilm bacteria. The authors found that LukAB and not NETosis determines the death of neutrophils exposed to *S. aureus* biofilms. Thus, leukocidins and the nuclease Nuc prevent neutrophil-mediated killing of *S. aureus* biofilms [96].

In addition to leukocidal toxins, *S. aureus* can secrete a variety of proteins that function as immune modulators and help this bacterium to elude the immune defenses by blocking the interactions of chemoattractants with the receptors expressed by neutrophils [6]. Sultan et al. have reported that peptides such as the earlier mentioned staphylococcal complement inhibitor (SCIN) and, to a lesser extent, the chemotaxis inhibitory protein of *Staphylococcus* (CHIPS, which binds C5aR and FPR1 on neutrophils) and the formyl peptide receptor-like 1 inhibitor (FLIPr, which blocks FPR1, FPR2, and FcRs) are transcribed as early as after 4 h of biofilm formation [97]. Sultan et al. observed that the levels of SCIN transcription during these early stages of biofilm formation in vitro were already sufficient to provide protection against complement activation [97], indicating the significant role that these immune modulators could play in the establishment of biofilms in vivo.

In recent years, it has emerged that, in different bacterial species, an important contribution to biofilm formation and stabilization has been associated with repurposed cytoplasmic proteins released in the outer space following bacterial cytolytic processes. These proteins are often referred to as moonlighting proteins, emphasizing their secondary functionalities acquired once they are in the extracellular space (moonlighting functions). Moonlighting proteins are involved in the architecture of bacterial biofilms; for instance, the members of the DNABII protein family, such as the integration host factor (IHF) and the histone-like nucleoid-associated protein (HU), and their interaction with eDNA have recently been reviewed in Campoccia et al. [14].

## 6. Teichoic Acids

Among the molecular components of the matrix of staphylococcal biofilms, there are teichoic acids. A strong impact of teichoic acids on adherence to biomaterial and biofilm formation has been observed in *S. aureus* and *E. faecalis* [80,98,99]. Wall teichoic acids (WTA) are charged glycopolymers mostly consisting of phosphodiester-linked polyol units, exclusively expressed in Gram-positive bacteria, and are major constituents (up to 60% of its dry weight) of the cell wall [100]. WTA and lipoteichoic acids (LTA), which are embedded in the membrane lipid bilayer, are involved in the interaction with many host receptors implicated in the process of colonization and infection, from initial adherence and activation of innate immunity to the induction of adaptive immune reactions [101]. In the complex biofilm architecture, negatively charged teichoic acid expressed on staphylococcal cells undergo electrostatic interaction with PIA, exhibiting a net positive charge. This interaction enables cell attachment and intercellular adhesion [102]. *S. aureus* LTA has an important role in recruiting inflammatory cells and in phagocytosis [103]. The role of *S. aureus* lipoteichoic acid in recruiting inflammatory cells has been investigated by Wagner et al. [104]. They demonstrated that LTA binds to and activates neutrophils, promoting the rapid upregulation of the adhesion protein CD18, the high-affinity IgG receptor (Fc-gamma receptor 1, CD64), the LPS-receptor (CD14), and the Fc receptors FcγRIIIa and FcγRIIIb (CD16), which together activate degranulation, phagocytosis, and oxidative burst; and the downregulation of CD62L (L-selectin) receptor [104]. Organization in biofilms did not prevent interactions of neutrophils with bacteria. The recognition of bacterial surface molecules, such as lipoteichoic acid, activates neutrophils, which, in the attempt to phagocytose biofilm, release cytotoxic mediators and proteolytic enzymes, leading to “frustrated phagocytosis” [105]. This phenomenon is analogously occurring with other professional phagocytes such as macrophages, when they are triggered and attempt to phagocytose and engulf foreign bodies larger than their size such as, for instance, implanted biomaterials. The authors propose that in post-traumatic osteomyelitis persistent neutrophil activation, “frustrated phagocytosis” and the lack of regulatory monocytes cause tissue degradation and osteolysis [11]. Additionally, WTA and LTA are both capable of triggering the activation of the lectin pathway of the complement, which involves, respectively, the binding of mannose-binding lectins (MBLs) or ficolin and the association of MBL-associated serine protease (MASP) complexes [6]. It should be said that WTA activation of the complement cascade through binding of MBLs would occur only in infants, who have not yet fully developed their adaptive immunity [106]. Conversely, in adults, anti-wall teichoic acid antibodies cause an inhibitory effect on serum MBL binding to WTA, and complement activation would occur through the classic pathway. Vice versa, LTA triggers the lectin pathway following the binding of ficolin [6].

In addition to all the above-described activities and staphylococcal (patho)physiologic functions, including bacterial adhesion and colonization, teichoic acids seem to act also in the protection from innate immune defense mechanisms, such as the action of antimicrobial peptides [107]. Notably, in *S. epidermidis*, the deletion of the gene that encodes for the first enzymatic step in the biosynthesis of wall teichoic acids stimulates an autolytic activity and impairs biofilm production [108].

Modifications to the Gram-positive bacterial cell wall play important roles in antibiotic resistance and pathogenesis [109]. Both WTA and LTA can be modified with positively charged d-alanine residues through a process that depends on the d-alanyl lipoteichoic acid (DLT) pathway. D-alanylation of LTA is common across Gram-positive bacteria. In *S. aureus*, d-alanylation is mediated by the *dlt* operon and increases, up to about 100-fold, bacterial resistance to the action of the enzyme phospholipase A_2_ group IIA [6,110,111,112], which is secreted by neutrophils and accumulates in inflammatory fluids. No effect of the d-alanylation of *S. aureus* (lipo)teichoic acids has been reported on the complement-mediated opsonization of the bacteria [111].

## 7. Lipids, Lipopolysaccharide (LPS), and Other Biofilm Components

Lipids represent a relatively low proportion of biofilm matrix mass. Their presence generally contributes to biofilm architecture conferring hydrophobicity. Nonetheless, there is very limited information available on their distribution, interaction with other molecules, and function in the extracellular space and in the biofilm matrix. Lipids also take part in the composition of more complex molecules such as the earlier mentioned lipoteichoic acid, lipoproteins (which play a crucial role in maintaining cellular integrity, establishing infections, and promoting biofilm formation [113]), and lipopolysaccharides (LPS).

A varying fraction of lipids within biofilms is associated with extracellular vesicles [114]. The various typologies and mechanisms of production of extracellular vesicles have recently been reviewed in [23]. Emerging evidence suggests that bacterial outer membrane vesicles (OMVs) contain differentially packaged short RNAs (sRNAs) with the potential to target host mRNA function and/or stability and could constitute a novel mechanism of host–pathogen interaction whereby pathogens such as *P. aeruginosa* hijack the host immune response [115].

Ciszek-Lenda et al. [116] reported that the biofilm of *P. aeruginosa* is a rich source of proinflammatory stimuli, including, in addition to eDNA and exopolysaccharides, the lipopolysaccharide (LPS). LPS would accumulate with the other biofilm components, representing, with eDNA, a main potent inducer of phagocyte hyperinflammation. Exopolysaccharides were found to stimulate cytokine production by neutrophils but only at concentrations higher than 30 µg/mL, never reached under in vitro culture conditions. On the contrary, marked stimulation of IL-6 secretion from neutrophils was observed at concentrations >100 ng/mL of LPS and >3 µg/mL of bacterial DNA. This in vitro study would provide support to the hypothesis that biofilm matrix components stimulate release of proinflammatory mediators and hyperinflammation, leading infiltrating phagocytes to become tissue-damaging cells, without the direct contact of neutrophils (and macrophages) with bacteria. Phagocytes exposed to the biofilm microenvironment of *P. aeruginosa* exhibit the proinflammatory secretory profiles referred to as N1/M1 phenotypes. As described above for opsonization, the interaction with the biofilm components would favor distant leukocyte activation and chronic inflammation with frustrated phagocytosis rather than effective killing of pathogens [116]. LPS recognition has been found to involve TLR4 and CD14. TLR 4 and myeloid differentiation factor 2 (MD-2) were found to form a heterodimer (the TLR4/MD-2 complex) capable of recognizing a common ‘pattern’ in structurally diverse LPS molecules. The TLR4/MD-2 complex is highly expressed on the surface of macrophages, monocytes, dendritic, and epithelial cells [117,118]. CD14 is a glycosylphosphatidylinositol-anchored protein expressed on the surface of monocytes, macrophages, and polymorphonuclear leukocytes that has long been known as an innate immune receptor binding complex of LPS with LPS binding protein (LBP). With TLR4, CD14 mediates the action of LPS on neutrophils. Glycosylphosphatidylinositol-anchored CD14 has been found to be the key player in LPS-induced human neutrophils priming for fMLP-triggered ROS production [119].

Vice versa, LPS endocytosed into the cytosol of host cells or cytosolic LPS produced by intracellular bacteria is recognized by cytosolic proteases caspase-4/11 and hosts guanylate binding proteins that are involved both in the assembly and activation of the NLRP3 inflammasome [118]. Interestingly, OMVs seem to play a critical role in enabling the cytosolic entry of LPS and, thus, caspase-11 activation in the presence of Gram-negative bacterial infections [120].

For many other lipids, the function in the bacterial biofilm and the type of interaction established with cells of the immune system remain largely unknown and warrant investigations.

## 8. A Glance at Biofilms Biodiversity

Bacteria within biofilms produce a wide variety of EPS during their growth. All the bacterial species involved in weaving the biofilm matrix contribute to this variety, which is further enriched by molecules derived from the tissues or fluids of the host. Stresses, temperature, light, and water, as well as the concentration and typology of molecules, salts, minerals, and nutrients in the *milieu*, influence the composition of the biofilm matrix [15]. In addition, it is increasingly emerging that in vitro-grown biofilms, with their extracellular matrices, are different from those that spontaneously form in living organisms. Indeed, a greater number and variety of microbial species can contribute to the formation of the in vivo biofilms. Moreover, the characteristics and composition of the different physiological environments reverberate on the molecular composition of the in vivo-formed biofilm matrices.

## 9. Conclusions

The biofilm matrix molecular components dynamically interact with each other. Interactions occur among eDNA, proteins, polysaccharides, and all other molecules in the biofilm matrix. For example, eDNA is a polyanionic polymer. Within *S. aureus* biofilms, both polysaccharides and many positively charged extracellular proteins have been reported to interact electrostatically with the negatively charged eDNA. The varied and complex interactions between the macromolecular components of the biofilm matrix strengthen the cohesion and stability of the biofilm architecture.

The study of the interactions between neutrophils and the individual components of the biofilm matrix is important and essential, while keeping us aware of the complexity of the biofilm matrix and its high level of diversity.

Indubitably, the biofilm matrix is a great little world still to be explored, not only by the audacious neutrophils but also by us.

## Figures and Tables

**Table 1:** Activity of different bacterial exopolysaccharides on immune response.

Exopolysaccharide	Species	Activity on Immune Response	Proinfl. **↑** Anti-Infl. **↓**	Ref.
**PIA** (Poly-β-(1,6)-*N*-acetylglucosamine)	*S. aureus*	Prevents antibody-independent opsonic killing by PMNs and complement	**↓**	[50]
	*S. epidermidis*	Protects bacteria from phagocytosis and bacterial killing by PMNs	**↓**	[48]
		Protects from AMPs (e.g., β-defensin 3, LL-37, and anionic dermcidin)	**↓**	[48]
		Resistance to opsonic PMN killing for diminished immunoglobulin and complement (C3b) deposition	**↓**	[53]
		Less PMNs activation and cytokine release	**↓**	[52]
		Strong complement activation	**↑** **↑**	[52]
**Psl** (Neutral mannose-rich polysaccharide)	*P. aeruginosa*	Reduce neutrophil phagocytosis Reduce ROS response (by limiting complement-mediated opsonization)	**↓**	[56]
		Purified Psl does not stimulate ROS production by the PMNs	**↓**	[61]
**Alginate** (Acetylated copolymer of 1–4 linked β-d-mannuronic acid and α-l-guluronic acid)	*P. aeruginosa*	Reduced phagocytosis by MØs and PMNs Scavenges ROS Inhibits chemotaxis	**↓**	[62,63,64,65]
**Pel**	*P. aeruginosa*	Possibly protecting bacteria from MØs phagocytosis	**↓**	[66]
		Purified Pel does not stimulate ROS production by the PMNs	**↓**	[61]
**Alginate/Psl-rich biofilms**	*P. aeruginosa*	Increased activation of the PMNs (ROS and degranulation)	**↑** **↑**	[55]
**Alginate/Pel-rich biofilms**	*P. aeruginosa*	Lower activation of the PMNs with respect to the Alginate/Psl combination	**↑**	[55]
**Polysaccharide capsule**	*K. kingae*	Protection from complement-mediated lysis Prevents neutrophil ROS production Interferes with neutrophil *K. kingae* binding	**↓**	[60]
**Exopolysaccharide** (Galactan, a galactofuranose homopolymer with formula [→5)-β-Galf-(1→]_n_ homopolymer [F3])	*K. kingae*	Protects against complement-mediated lysis Evasion of neutrophil-mediated killing Blocks neutrophil phagocytosis	**↓**	[59,60]
**Exopolysaccharide** (Strain-dependent mixture of different polysaccharides)	*Burkholderia cepacia*	Inhibits PMNs chemotaxis Inhibits PMNs ROS production	**↓**	[67,68]

## Data Availability

Data are contained within the article.

## References

[1] Poultsides L.A., Liaropoulos L.L., Malizos K.N. (2010). The socioeconomic impact of musculoskeletal infections. J. Bone Jt. Surg. Am..

[2] Campoccia D., Montanaro L., Arciola C.R. (2013). A review of the clinical implications of anti-infective biomaterials and infection-resistant surfaces. Biomaterials.

[3] Arciola C.R., Campoccia D., Montanaro L. (2018). Implant infections: Adhesion, biofilm formation and immune evasion. Nat. Rev. Microbiol..

[4] Campoccia D., Montanaro L., Ravaioli S., Cangini I., Testoni F., Visai L., Arciola C.R. (2018). New Parameters to Quantitatively Express the Invasiveness of Bacterial Strains from Implant-Related Orthopaedic Infections into Osteoblast Cells. Materials.

[5] De Mesy Bentley K.L., Trombetta R., Nishitani K., Bello-Irizarry S.N., Ninomiya M., Zhang L., Chung H.L., McGrath J.L., Daiss J.L., Awad H.A. (2017). Evidence of *Staphylococcus aureus* Deformation, Proliferation, and Migration in Canaliculi of Live Cortical Bone in Murine Models of Osteomyelitis. J. Bone Miner. Res. Off. J. Am. Soc. Bone Miner. Res..

[6] De Vor L., Rooijakkers S.H.M., van Strijp J.A.G. (2020). Staphylococci evade the innate immune response by disarming neutrophils and forming biofilms. FEBS Lett..

[7] Cruz A.R., Boer M.A.D., Strasser J., Zwarthoff S.A., Beurskens F.J., de Haas C.J.C., Aerts P.C., Wang G., de Jong R.N., Bagnoli F. (2021). Staphylococcal protein A inhibits complement activation by interfering with IgG hexamer formation. Proc. Natl. Acad. Sci. USA.

[8] Cruz A.R., Bentlage A.E.H., Blonk R., de Haas C.J.C., Aerts P.C., Scheepmaker L.M., Bouwmeester I.G., Lux A., van Strijp J.A.G., Nimmerjahn F. (2022). Toward Understanding How Staphylococcal Protein A Inhibits IgG-Mediated Phagocytosis. J. Immunol..

[9] Masters E.A., Trombetta R.P., de Mesy Bentley K.L., Boyce B.F., Gill A.L., Gill S.R., Nishitani K., Ishikawa M., Morita Y., Ito H. (2019). Evolving concepts in bone infection: Redefining “biofilm”, “acute vs. chronic osteomyelitis”, “the immune proteome” and “local antibiotic therapy”. Bone Res..

[10] Campoccia D., Mirzaei R., Montanaro L., Arciola C.R. (2019). Hijacking of immune defences by biofilms: A multifront strategy. Biofouling.

[11] Arciola C.R., Ravaioli S., Mirzaei R., Dolzani P., Montanaro L., Daglia M., Campoccia D. (2023). Biofilms in periprosthetic orthopedic infections seen through the eyes of neutrophils. How Can We Help Neutrophils?. Int. J. Mol. Sci..

[12] Tomlinson K.L., Riquelme S.A., Baskota S.U., Drikic M., Monk I.R., Stinear T.P., Lewis I.A., Prince A.S. (2023). *Staphylococcus aureus* stimulates neutrophil itaconate production that suppresses the oxidative burst. Cell Rep..

[13] Heim C.E., Bosch M.E., Yamada K.J., Aldrich A.L., Chaudhari S.S., Klinkebiel D., Gries C.M., Alqarzaee A.A., Li Y., Thomas V.C. (2020). Lactate production by *Staphylococcus aureus* biofilm inhibits HDAC11 to reprogramme the host immune response during persistent infection. Nat. Microbiol..

[14] Campoccia D., Montanaro L., Arciola C.R. (2021). Extracellular DNA (eDNA). A Major Ubiquitous Element of the Bacterial Biofilm Architecture. Int. J. Mol. Sci..

[15] Flemming H.C., van Hullebusch E.D., Neu T.R., Nielsen P.H., Seviour T., Stoodley P., Wingender J., Wuertz S. (2023). The biofilm matrix: Multitasking in a shared space. Nat. Rev. Microbiol..

[16] Ravaioli S., Campoccia D., Speziale P., Pietrocola G., Zatorska B., Maso A., Presterl E., Montanaro L., Arciola C.R. (2020). Various biofilm matrices of the emerging pathogen *Staphylococcus lugdunensis*: Exopolysaccharides, proteins, eDNA and their correlation with biofilm mass. Biofouling.

[17] Harris L.G., Murray S., Pascoe B., Bray J., Meric G., Mageiros L., Wilkinson T.S., Jeeves R., Rohde H., Schwarz S. (2016). Biofilm Morphotypes and Population Structure among *Staphylococcus epidermidis* from Commensal and Clinical Samples. PLoS ONE.

[18] Nguyen H.T.T., Nguyen T.H., Otto M. (2020). The staphylococcal exopolysaccharide PIA—Biosynthesis and role in biofilm formation, colonization, and infection. Comput. Struct. Biotechnol. J..

[19] Ozinsky A., Underhill D.M., Fontenot J.D., Hajjar A.M., Smith K.D., Wilson C.B., Schroeder L., Aderem A. (2000). The repertoire for pattern recognition of pathogens by the innate immune system is defined by cooperation between toll-like receptors. Proc. Natl. Acad. Sci. USA.

[20] Weiß E., Schlatterer K., Beck C., Peschel A., Kretschmer D. (2020). Formyl-Peptide Receptor Activation Enhances Phagocytosis of Community-Acquired Methicillin-Resistant *Staphylococcus aureus*. J. Infect. Dis..

[21] Okshevsky M., Regina V.R., Meyer R.L. (2015). Extracellular DNA as a target for bio-film control. Curr. Opin. Biotechnol..

[22] Whitchurch C.B., Tolker-Nielsen T., Ragas P.C., Mattick J.S. (2002). Extracellular DNA required for bacterial biofilm formation. Science.

[23] Campoccia D., Montanaro L., Arciola C.R. (2021). Tracing the origins of extracellular DNA in bacterial biofilms: Story of death and predation to community benefit. Biofouling.

[24] Rajendran N.B., Eikmeier J., Becker K., Hussain M., Peters G., Heilmann C. (2015). Important contribution of the novel locus comEB to extracellular DNA-dependent *Staphylococcus lugdunensis* biofilm formation. Infect. Immun..

[25] Alhede M., Alhede M., Qvortrup K., Kragh K.N., Jensen P.Ø., Stewart P.S., Bjarnsholt T. (2020). The origin of extracellular DNA in bacterial biofilm infections in vivo. Pathog. Dis..

[26] Montanaro L., Poggi A., Visai L., Ravaioli S., Campoccia D., Speziale P., Arciola C.R. (2011). Extracellular DNA in biofilms. Int. J. Artif. Organs.

[27] Hornung V., Latz E. (2010). Intracellular DNA recognition. Nat. Rev. Immunol..

[28] Hemmi H., Takeuchi O., Kawai T., Kaisho T., Sato S., Sanjo H., Matsumoto M., Hoshino K., Wagner H., Takeda K. (2000). A Toll-like receptor recognizes bac-terial DNA. Nature.

[29] Bauer S., Kirschning C.J., Häcker H., Redecke V., Hausmann S., Akira S., Wagner H. (2001). Lipford GB. Human TLR9 confers responsiveness to bacterial DNA via species-specific CpG motif recognition. Proc. Natl. Acad. Sci. USA.

[30] Kawai T., Akira S. (2010). The role of pattern-recognition receptors in innate immunity: Update on Toll-like receptors. Nat. Immunol..

[31] Tursi S.A., Tükel Ç. (2018). Curli-Containing Enteric Biofilms Inside and Out: Matrix Composition, Immune Recognition, and Disease Implications. Microbiol. Mol. Biol. Rev. MMBR.

[32] Tursi S.A., Lee E.Y., Medeiros N.J., Lee M.H., Nicastro L.K., Buttaro B., Gallucci S., Wilson R.P., Wong G.C.L., Tükel Ç. (2017). Bacterial amyloid curli acts as a carrier for DNA to elicit an autoimmune response via TLR2 and TLR9. PLoS Pathog..

[33] Fuxman Bass J.I., Russo D.M., Gabelloni M.L., Geffner J.R., Giordano M., Catalano M., Zorreguieta A., Trevani A.S. (2010). Extracellular DNA: A Major Proinflammatory Component of *Pseudomonas aeruginosa* Biofilms. J. Immunol..

[34] Bass J.I.F., Gabelloni M.L., Alvarez M.E., Vermeulen M.E., Russo D.M., Zorreguieta A., Geffner J.R., Trevani A.S. (2008). Characterization of bacterial DNA binding to human neutrophil surface. Lab. Investig..

[35] Latz E., Verma A., Visintin A., Gong M., Sirois C.M., Klein D.C., Monks B.G., McKnight C.J., Lamphier M.S., Duprex W.P. (2007). Ligand-induced conformational changes allosterically activate Toll-like receptor 9. Nat. Immunol..

[36] Campoccia D., Baldassarri L., Pirini V., Ravaioli S., Montanaro L., Arciola C.R. (2008). Molecular epidemiology of *Staphylococcus aureus* from implant orthopaedic infections: Ribotypes, agr polymorphism, leukocidal toxins and antibiotic resistance. Biomaterials.

[37] Vuong C., Kocianova S., Voyich J.M., Yao Y., Fischer E.R., DeLeo F.R., Otto M. (2004). A crucial role for exopolysaccharide modification in bacterial biofilm formation, immune evasion, and virulence. J. Biol. Chem..

[38] Mack D., Fischer W., Krokotsch A., Leopold K., Hartmann R., Egge H., Laufs R. (1996). The intercellular adhesin involved in biofilm accumulation of *Staphylococcus epidermidis* is a linear beta-1,6-linked glucosaminoglycan: Purification and structural analysis. J. Bacteriol..

[39] Ziebuhr W., Krimmer V., Rachid S., Lössner I., Götz F., Hacker J. (1999). A novel mechanism of phase variation of virulence in *Staphylococcus epidermidis*: Evidence for control of the polysaccharide intercellular adhesin synthesis by alternating insertion and excision of the insertion sequence element IS256. Mol. Microbiol..

[40] Arciola C.R., Gamberini S., Campoccia D., Visai L., Speziale P., Baldassarri L., Montanaro L. (2005). A multiplex PCR method for the detection of all five individual genes of *ica* locus in Staphylococcus epidermidis. A survey on 400 clinical isolates from prosthesis-associated infections. J. Biomed. Mater. Res. A.

[41] Arciola C.R., Campoccia D., Gamberini S., Rizzi S., Donati M.E., Baldassarri L., Montanaro L. (2004). Search for the insertion element IS256 within the *ica* locus of *Staphylococcus epidermidis* clinical isolates collected from biomaterial-associated infections. Biomaterials.

[42] Kozitskaya S., Cho S.H., Dietrich K., Marre R., Naber K., Ziebuhr W. (2004). The bacterial insertion sequence element IS256 occurs preferentially in nosocomial *Staphylococcus epidermidis* isolates: Association with biofilm formation and resistance to aminoglycosides. Infect. Immun..

[43] Perez M., Calles-Enríquez M., del Rio B., Ladero V., Martín M.C., Fernández M., Alvarez M.A. (2015). IS*256* abolishes gelatinase activity and biofilm formation in a mutant of the nosocomial pathogen *Enterococcus faecalis* V583. Can. J. Microbiol..

[44] Brooks J.L., Jefferson K.K. (2014). Phase variation of poly-N-acetylglucosamine expression in *Staphylococcus aureus*. PLoS Pathog..

[45] Glynn A.A., O’Donnell S.T., Molony D.C., Sheehan E., McCormack D.J., O’Gara J.P. (2009). Hydrogen peroxide induced repression of icaADBC transcription and biofilm development in *Staphylococcus epidermidis*. J. Orthop. Res..

[46] Wu Y., Wu Y., Zhu T., Han H., Liu H., Xu T., François P., Fischer A., Bai L., Götz F. (2015). *Staphylococcus epidermidis* SrrAB regulates bacterial growth and biofilm formation differently under oxic and microaerobic conditions. J. Bacteriol..

[47] Lin M.H., Shu J.C., Huang H.Y., Cheng Y.C. (2012). Involvement of iron in biofilm formation by *Staphylococcus aureus*. PLoS ONE.

[48] Vuong C., Voyich J.M., Fischer E.R., Braughton K.R., Whitney A.R., DeLeo F.R., Otto M. (2004). Polysaccharide intercellular ad-hesin (PIA) protects *Staphylococcus epidermidis* against major components of the human innate immune system. Cell. Microbiol..

[49] Cerca N., Jefferson K.K., Oliveira R., Pier G.B., Azeredo J. (2006). Comparative antibody-mediated phagocytosis of *Staphylococcus epidermidis* cells grown in a biofilm or in the planktonic state. Infect. Immun..

[50] Kropec A., Maira-Litran T., Jefferson K.K., Grout M., Cramton S.E., Götz F., Goldmann D.A., Pier G.B. (2005). Poly-N-acetylglucosamine production in *Staphylococcus aureus* is essen-tial for virulence in murine models of systemic infection. Infect. Immun..

[51] Le K.Y., Park M.D., Otto M. (2018). Immune Evasion Mechanisms of *Staphylococcus epidermidis* Biofilm Infection. Front. Microbiol..

[52] Fredheim E.G., Granslo H.N., Flægstad T., Figenschau Y., Rohde H., Sadovskaya I., Mollnes T.E., Klingenberg C. (2011). *Staphylococcus epidermidis* polysaccharide inter-cellular adhesin activates complement. FEMS Immunol. Med. Microbiol..

[53] Kristian S.A., Birkenstock T.A., Sauder U., Mack D., Götz F., Landmann R. (2008). Biofilm formation induces C3a release and protects *Staphylococcus epidermidis* from IgG and complement deposition and from neutrophil-dependent killing. J. Infect. Dis..

[54] Malhotra S., Hayes D., Wozniak D.J. (2019). Cystic Fibrosis and Pseudomonas ae-ruginosa: The Host-Microbe Interface. Clin. Microbiol. Rev..

[55] Rybtke M., Jensen P.Ø., Nielsen C.H., Tolker-Nielsen T. (2020). The Extracellular Polysaccharide Matrix of *Pseudomonas aeruginosa* Biofilms Is a Determinant of Polymorphonuclear Leukocyte Responses. Infect. Immun..

[56] Mishra M., Byrd M.S., Sergeant S., Azad A.K., Parsek M.R., McPhail L., Schlesinger L.S., Wozniak D.J. (2012). *Pseudomonas aeruginosa* Psl polysaccharide reduces neutrophil phagocytosis and the oxidative response by limiting complement-mediated opsonization. Cell. Microbiol..

[57] Moshirabadi A., Razi M., Arasteh P., Sarzaeem M.M., Ghaffari S., Aminiafshar S., Hos-seinian Khosroshahy K., Sheikholeslami F.M. (2019). Polymerase Chain Reaction Assay Using the Restriction Fragment Length Polymorphism Technique in the Detection of Prosthetic Joint Infections: A Multi-Centered Study. J. Arthroplast..

[58] Wensley K., McClelland D., Grocott N., Manoharan G., Desai S. (2023). Case Report: Kingella kingae causing prosthetic joint infection in an adult. Access Microbiol..

[59] Muñoz V.L., Porsch E.A., St Geme J.W. (2018). Kingella kingae Surface Polysaccharides Promote Resistance to Human Serum and Virulence in a Juvenile Rat Model. Infect. Immun..

[60] Muñoz V.L., Porsch E.A., St Geme J.W. (2019). Kingella kingae Surface Polysaccharides Promote Resistance to Neutrophil Phagocytosis and Killing. mBio.

[61] Pestrak M.J., Chaney S.B., Eggleston H.C., Dellos-Nolan S., Dixit S., Mathew-Steiner S.S., Roy S., Parsek M.R., Sen C.K., Wozniak D.J. (2018). *Pseudomonas aeruginosa* rugose small-colony variants evade host clearance, are hyper-inflammatory, and persist in multiple host environments. PLoS Pathog..

[62] Cabral D.A., Loh B.A., Speert D.P. (1987). Mucoid *Pseudomonas aeruginosa* resists nonopsonic phagocytosis by human neutrophils and macrophages. Pediatr. Res..

[63] Leid J.G., Willson C.J., Shirtliff M.E., Hassett D.J., Parsek M.R., Jeffers A.K. (2005). The exopolysaccharide alginate protects *Pseudomonas aeruginosa* biofilm bacteria from IFN-gamma-mediated macrophage killing. J. Immunol..

[64] Learn D.B., Brestel E.P., Seetharama S. (1987). Hypochlorite scavenging by *Pseudomonas aeruginosa* alginate. Infect. Immun..

[65] Simpson J.A., Smith S.E., Dean R.T. (1989). Scavenging by alginate of free radicals released by macrophages. Free Radic. Biol. Med..

[66] Malone J.G., Jaeger T., Spangler C., Ritz D., Spang A., Arrieumerlou C., Kaever V., Landmann R., Jenal U. (2010). YfiBNR mediates cyclic di-GMP dependent small colony variant formation and persistence in *Pseudomonas aeruginosa*. PLoS Pathog..

[67] Bylund J., Burgess L.A., Cescutti P., Ernst R.K., Speert D.P. (2006). Exopolysaccharides from Burkholderia cenocepacia inhibit neutrophil chemotaxis and scavenge reactive oxygen species. J. Biol. Chem..

[68] Conway B.A., Chu K.K., Bylund J., Altman E., Speert D.P. (2004). Production of exopolysaccharide by Burkholderia cenocepacia results in altered cell-surface interactions and altered bacterial clearance in mice. J. Infect. Dis..

[69] Gil C., Solano C., Burgui S., Latasa C., García B., Toledo-Arana A., Lasa I., Valle J. (2014). Biofilm matrix exoproteins induce a protective immune response against *Staphylococcus aureus* biofilm infection. Infect. Immun..

[70] Meyle E., Brenner-Weiss G., Obst U., Prior B., Hänsch G.M. (2012). Immune defense against *S. epidermidis* biofilms: Components of the extracellular polymeric substance activate distinct bactericidal mechanisms of phagocytic cells. Int. J. Artif. Organs.

[71] Maurer S., Fouchard P., Meyle E., Prior B., Hänsch G.M., Dapunt U. (2015). Activation of neutrophils by the extracellular poly-meric substance of *S. epidermidis* biofilms is mediated by the bacterial heat shock protein groel. J. Biotechnol. Biomater..

[72] Dapunt U., Gaida M.M., Meyle E., Prior B., Hänsch G.M. (2016). Activation of phagocytic cells by staphylococcus epidermidis biofilms: Effects of ex-tracellular matrix proteins and the bacterial stress protein GroEL on netosis and MRP-14 release. Pathog. Dis..

[73] Stapels D.A., Ramyar K.X., Bischoff M., Von Kockritz-Blickwede M., Milder F.J., Ruyken M., Eisenbeis J., McWhorter W.J., Herrmann M., van Kessel K.P. (2014). *Staphylococcus aureus* secretes a unique class of neutrophil serine protease inhibitors. Proc. Natl. Acad. Sci. USA.

[74] Thompson K.M., Abraham N., Jefferson K.K. (2010). *Staphylococcus aureus* extra-cellular adherence protein contributes to biofilm formation in the presence of serum. FEMS Microbiol. Lett..

[75] Eisenbeis J., Saffarzadeh M., Peisker H., Jung P., Thewes N., Preissner K.T., Herrmann M., Molle V., Geisbrecht B.V., Jacobs K. (2018). The *Staphylococcus aureus* Extra-cellular Adherence Protein Eap Is a DNA Binding Protein Capable of Blocking Neutrophil Extracellular Trap Formation. Front. Cell. Infect. Microbiol..

[76] Arciola C.R., Campoccia D., Speziale P., Montanaro L., Costerton J.W. (2012). Biofilm formation in *Staphylococcus* implant infections. A review of molecular mechanisms and implications for biofilm-resistant materials. Biomaterials.

[77] Cucarella C., Solano C., Valle J., Amorena B., Lasa I., Penadés J.R. (2001). Bap, a *Staphylococcus aureus* surface protein involved in biofilm formation. J. Bacteriol..

[78] Speziale P., Pietrocola G., Foster T.J., Geoghegan J.A. (2014). Protein-based biofilm matrices in Staphylococci. Front. Cell. Infect. Microbiol..

[79] Foster T.J., Geoghegan J.A., Ganesh V.K., Höök M. (2014). Adhesion, invasion and evasion: The many functions of the surface proteins of *Staphylococcus aureus*. Nat. Rev. Microbiol..

[80] Fabretti F., Theilacker C., Baldassarri L., Kaczynski Z., Kropec A., Holst O., Huebner J. (2006). Alanine esters of enterococcal lipoteichoic acid play a role in biofilm formation and resistance to antimicrobial peptides. Infect. Immun..

[81] Resch A., Rosenstein R., Nerz C., Götz F. (2005). Differential gene expression profiling of *Staphylococcus aureus* cultivated under biofilm and planktonic conditions. Appl. Environ. Microbiol..

[82] Paharik A.E., Kotasinska M., Both A., Hoang T.N., Büttner H., Roy P., Fey P.D., Horswill A.R., Rohde H. (2017). The metalloprotease SepA governs processing of accumulation-associated pro-tein and shapes intercellular adhesive surface properties in *Staphylococcus epidermidis*. Mol. Microbiol..

[83] Gómez-Alonso I.S., Martínez-García S., Betanzos-Cabrera G., Juárez E., Sara-bia-León M.C., Herrera M.T., Gómez-Chávez F., Sanchez-Torres L., Rodríguez-Martínez S., Cancino-Diaz M.E. (2022). Low Concentration of the Neutrophil Proteases Cathep-sin G, Cathepsin B, Proteinase-3 and Metalloproteinase-9 Induce Biofilm Formation in Non-Biofilm-Forming *Staphylococcus epidermidis* Isolates. Int. J. Mol. Sci..

[84] Conrady D.G., Wilson J.J., Herr A.B. (2013). Structural basis for Zn^2+^-dependent intercellular adhesion in staphylococcal biofilms. Proc. Natl. Acad. Sci. USA.

[85] Vergara-Irigaray M., Valle J., Merino N., Latasa C., García B., Ruiz de Los Mozos I., Solano C., Toledo-Arana A., Penadés J.R., Lasa I. (2009). Relevant role of fibronectin-binding proteins in *Staphylococcus aureus* biofilm-associated foreign-body infections. Infect. Immun..

[86] Burke F.M., Di Poto A., Speziale P., Foster T.J. (2011). The A domain of fibronectin-binding protein B of *Staphylococcus aureus* contains a novel fibronectin binding site. FEBS J..

[87] Montanaro L., Testoni F., Poggi A., Visai L., Speziale P., Arciola C.R. (2011). Emerging pathogenetic mechanisms of the implant-related osteomyelitis by *Staphylococcus aureus*. Int. J. Artif. Organs.

[88] Claro T., Widaa A., McDonnell C., Foster T.J., O’Brien F.J., Kerrigan S.W. (2013). *Staphylococcus aureus* protein A binding to osteoblast tumour necrosis factor receptor 1 results in activation of nuclear factor kappa B and release of interleukin-6 in bone infection. Microbiology.

[89] Urish K.L., Cassat J.E. (2020). *Staphylococcus aureus* Osteomyelitis: Bone, Bugs, and Surgery. Infect. Immun..

[90] Schwartz K., Syed A.K., Stephenson R.E., Rickard A.H., Boles B.R. (2012). Functional amyloids composed of phenol soluble modulins stabilize *Staphylococcus aureus* biofilms. PloS Pathog..

[91] Le K.Y., Villaruz A.E., Zheng Y., He L., Fisher E.L., Nguyen T.H., Ho T.V., Yeh A.J., Joo H.S., Cheung G.Y.C. (2019). Role of Phenol-Soluble Modulins in *Staphylococcus epidermidis* Biofilm Formation and Infection of Indwelling Medical Devices. J. Mol. Biol..

[92] Zheng Y., Joo H.S., Nair V., Le K.Y., Otto M. (2018). Do amyloid structures formed by *Staphylococcus aureus* phenol-soluble modulins have a biological function?. Int. J. Med. Microbiol..

[93] Salinas N., Povolotsky T.L., Landau M., Kolodkin-Gal I. (2020). Emerging Roles of Functional Bacterial Amyloids in Gene Regulation, Toxicity, and Immunomodulation. Microbiol. Mol. Biol. Rev..

[94] Spaan A.N., van Strijp J.A.G., Torres V.J. (2017). Leukocidins: Staphylococcal bi-component pore-forming toxins find their receptors. Nat. Rev. Microbiol..

[95] Bhattacharya M., Berends E.T.M., Chan R., Schwab E., Roy S., Sen C.K., Torres V.J., Wozniak D.J. (2018). *Staphylococcus aureus* biofilms release leukocidins to elicit extracel-lular trap formation and evade neutrophil-mediated killing. Proc. Natl. Acad. Sci. USA.

[96] Bhattacharya M., Berends E.T.M., Zheng X., Hill P.J., Chan R., Torres V.J., Wozniak D.J. (2020). Leukocidins and the Nuclease Nuc Prevent Neutrophil-Mediated Killing of *Staphylococcus aureus* Biofilms. Infect. Immun..

[97] Sultan A.R., Swierstra J.W., Lemmens-den Toom N.A., Snijders S.V., Hansenová Maňásková S., Verbon A., van Wamel W.J.B. (2018). Production of Staphylococcal Complement Inhibitor (SCIN) and Other Immune Modulators during the Early Stages of *Staphylococcus aureus* Biofilm Formation in a Mammalian Cell Culture Medium. Infect. Immun..

[98] Xia G., Kohler T., Peschel A. (2010). The wall teichoic acid and lipoteichoic acid polymers of *Staphylococcus aureus*. Int. J. Med. Microbiol..

[99] Spengler C., Nolle F., Thewes N., Wieland B., Jung P., Bischoff M., Jacobs K. (2021). Using knock-out mutants to investigate the adhesion of *Staphylococcus aureus* to abiotic surfaces. Int. J. Mol. Sci..

[100] Wu X., Han J., Gong G., Koffas M.A.G., Zha J. (2021). Wall teichoic acids: Physiology and applications. FEMS Microbiol. Rev..

[101] Weidenmaier C., Peschel A. (2008). Teichoic acids and related cell-wall glycopolymers in Gram-positive physiology and host interactions. Nat. Rev. Microbiol..

[102] Formosa-Dague C., Feuillie C., Beaussart A., Derclaye S., Kucharikova S., Lasa I., Van Dijck P., Dufrene Y.F. (2016). Sticky matrix: Adhesion mechanism of the staphylococcal polysaccharide intercellular adhesin. ACS Nano.

[103] Kawai T., Akira S. (2011). Toll-like receptors and their crosstalk with other innate receptors in infection and immunity. Immunity.

[104] Wagner C., Obst U., Hänsch G.M. (2005). Implant-associated posttraumatic osteomyelitis: Collateral damage by local host defense?. Int. J. Artif. Organs.

[105] Wagner C., Kondella K., Bernschneider T., Heppert V., Wentzensen A., Hänsch G.M. (2003). Post-traumatic osteomyelitis: Analysis of inflammatory cells recruited into the site of infection. Shock.

[106] Park K.H., Kurokawa K., Zheng L., Jung D.J., Tateishi K., Jin J.O., Ha N.C., Kang H.J., Matsushita M., Kwak J.Y. (2010). Human serum mannose-binding lectin senses wall teichoic acid Glycopolymer of *Staphylococcus aureus*, which is restricted in infancy. J. Biol. Chem..

[107] Assoni L., Milani B., Carvalho M.R., Nepomuceno L.N., Waz N.T., Guerra M.E.S., Converso T.R., Darrieux M. (2020). Resistance Mechanisms to Antimicrobial Peptides in Gram-Positive Bacteria. Front. Microbiol..

[108] Holland L.M., Conlon B., O’Gara J.P. (2011). Mutation of tagO reveals an essential role for wall teichoic acids in *Staphylococcus epidermidis* biofilm development. Microbiology.

[109] Wood B.M., Santa Maria J.P., Matano L.M., Vickery C.R., Walker S. (2018). A partial reconstitution implicates DltD in catalyzing lipoteichoic acid d-alanylation. J. Biol. Chem..

[110] Koprivnjak T., Peschel A., Gelb M.H., Liang N.S., Weiss J.P. (2002). Role of charge properties of bacterial envelope in bactericidal action of human group IIA phospholipase A2 against *Staphylococcus aureus*. J. Biol. Chem..

[111] Hunt C.L., Nauseef W.M., Weiss J.P. (2006). Effect of D-alanylation of (lipo)teichoic acids of *Staphylococcus aureus* on host secretory phospholipase A2 action before and after phagocytosis by human neutrophils. J. Immunol..

[112] Nevalainen T.J., Graham G.G., Scott K.F. (2008). Antibacterial actions of secreted phospholipases A2. Review. Biochim. Biophys. Acta.

[113] Liu S., Lu H., Zhang S., Shi Y., Chen Q. (2022). Phages against Pathogenic Bacterial Biofilms and Biofilm-Based Infections: A Review. Pharmaceutics.

[114] Henriquez T., Falciani C. (2023). Extracellular Vesicles of Pseudomonas: Friends and Foes. Antibiotics.

[115] Koeppen K., Hampton T.H., Jarek M., Scharfe M., Gerber S.A., Mielcarz D.W., Demers E.G., Dolben E.L., Hammond J.H., Hogan D.A. (2016). A Novel Mechanism of Host-Pathogen Interaction through sRNA in Bacterial Outer Membrane Vesicles. PLoS Pathog..

[116] Ciszek-Lenda M., Strus M., Walczewska M., Majka G., Machul-Żwirbla A., Mikołajczyk D., Górska S., Gamian A., Chain B., Marcinkiewicz J. (2019). *Pseudomonas aeruginosa* biofilm is a potent inducer of phagocyte hyperinflammation. Inflamm. Res..

[117] Park B.S., Song D.H., Kim H.M., Choi B.S., Lee H., Lee J.O. (2009). The structural basis of lipopolysaccharide recognition by the TLR4-MD-2 complex. Nature.

[118] Zamyatina A., Heine H. (2020). Lipopolysaccharide Recognition in the Crossroads of TLR4 and Caspase-4/11 Mediated Inflammatory Pathways. Front. Immunol..

[119] Kabanov D.S., Vwedenskaya O.Y., Fokina M.A., Morozova E.M., Grachev S.V., Prokhorenko I.R. (2019). Impact of CD14 on Reactive Oxygen Species Production from Human Leukocytes Primed by Escherichia coli Lipopolysaccharides. Oxid. Med. Cell. Longev..

[120] Vanaja S.K., Russo A.J., Behl B., Banerjee I., Yankova M., Deshmukh S.D., Rathinam V.A.K. (2016). Bacterial Outer Membrane Vesicles Mediate Cytosolic Localization of LPS and Caspase-11 Activation. Cell.

